# A Comparison of the Environmental Impact of Different AOPs: Risk Indexes

**DOI:** 10.3390/molecules20010503

**Published:** 2014-12-31

**Authors:** Jaime Giménez, Bernardí Bayarri, Óscar González, Sixto Malato, José Peral, Santiago Esplugas

**Affiliations:** 1 Departament d’Enginyeria Química, Universitat de Barcelona, C/Martí i Franquès 1, Barcelona 08028, Spain; E-Mails: bbayarri@ub.edu (B.B.); oscar@fegon.es (O.G.); santi.esplugas@ub.edu (S.E.); 2 Plataforma Solar de Almería (CIEMAT), Carretera de Senes, km 4, Tabernas, Almería 04200, Spain; E-Mail: sixto.malato@psa.es; 3 Departament de Química, Edifici Cn, Universitat Autònoma de Barcelona, Bellaterra, Cerdanyola del Vallès 08193, Spain; E-Mail: Jose.Peral@uab.cat

**Keywords:** AOPs environmental impact, risk assessment, environmental risk indexes, AOPs comparison, sustainability

## Abstract

Today, environmental impact associated with pollution treatment is a matter of great concern. A method is proposed for evaluating environmental risk associated with Advanced Oxidation Processes (AOPs) applied to wastewater treatment. The method is based on the type of pollution (wastewater, solids, air or soil) and on materials and energy consumption. An Environmental Risk Index (E), constructed from numerical criteria provided, is presented for environmental comparison of processes and/or operations. The Operation Environmental Risk Index (E_Oi_) for each of the unit operations involved in the process and the Aspects Environmental Risk Index (E_Aj_) for process conditions were also estimated. Relative indexes were calculated to evaluate the risk of each operation (E/NOP) or aspect (E/NAS) involved in the process, and the percentage of the maximum achievable for each operation and aspect was found. A practical application of the method is presented for two AOPs: photo-Fenton and heterogeneous photocatalysis with suspended TiO_2_ in Solarbox. The results report the environmental risks associated with each process, so that AOPs tested and the operations involved with them can be compared.

## 1. Introduction

Sustainability is one of today’s most important goals in any human activity, and decreased environmental impacts must be a basic objective for its achievement. Therefore, many businesses are implementing environment management systems following such standards as the ISO 14001 [1] or EMAS [2], which are the starting point for pollution control.

There are several different methods for evaluating environmental impact. Life Cycle Assessment (LCA) is one of the most widely used. LCA includes four steps: goal and scope definition, inventory analysis, Life Cycle Impact Assessment (LCIA) and interpretation of results. In the first step (goal and scope), the limits of the system and the focus of the analysis are defined. Inventory analysis involves compiling an inventory of raw materials, energy consumption and pollutants production. In the LCIA step, the data from the inventory are assigned to different impact categories and characterized according to the corresponding factors. These impact categories depend on whether midpoint methods [3,4], defining only impacts in categories such as ozone depletion, climate change, *etc.*, or endpoint methods [5,6], which analyze final impacts on human health, environment or resources consumption, are used.

Environmental consequences can also be analysed using an environmental risk index, as provided for in Directive 96/82/EC [7], which is to be replaced as of June 1, 2015, by Directive 2012/18/EU [8]. It is based on the analysis of four parameters: risk sources (analysis of possible accidents including hazardous substances involved), primary control systems (preventive and protective measures to reduce the consequences of an accident, for example, retention ponds), transport systems (pollutant concentration profile in the area affected by a spill), and sensitive receptors (agricultural areas, protected species, monuments, *etc.*, present in the area affected by a spill). With these factors, the environmental consequences index can be calculated, which when combined with the probability of an accident, results in an environmental risk index.

A third method of environmental analysis is based on Directive 2004/35/CE [9]. This Directive proposes a methodology very similar to the one used for risk evaluation in industrial safety. The first step is the identification of hazards and possible environmental accidents by techniques such as the event tree. The second step is modelling the accidents and evaluating their consequences, considering the setting where they occur. Then risk is evaluated by including their probability, estimated, for instance, by tree fault analysis. Finally, an economic assessment is made of damage caused by the accidents and the cost of recovery of affected areas.

Finally, the methodology (Arteche *et al.* [10]) selected for the estimation of environmental impact in this study is based on application of risk indexes [11]. The first stage is identification of the steps in the processes under study in three different situations: normal, abnormal and emergency operations. The environmental impact is evaluated for each considering two aspects, pollution (wastewater, solids, air or soil) and consumption (raw materials, water, energy). Risk assessment is based on frequency (probability), hazard and amount (volume). Numerical criteria are provided to assign a value to the environmental risk, thus enabling comparison of the environmental impact of processes and/or operations. The numerical criteria proposed by Arteche *et al.* [10] were updated and adapted to the particular cases studied, which were laboratory or small pilot plant operations, and current legislation. The values found in this paper are therefore noticeably different from those originally proposed by Arteche *et al.* [10]. The final result is the Global Environmental Risk Index (E), which gives an overview of the environmental priority of each activity related to other analogous activities. Its evaluation is the main purpose of this paper. Other related indexes are also evaluated for comparison of the various process operations and aspects involved. This method is amply explained in the following sections, and a practical application is developed for two AOPs, photo-Fenton and heterogeneous photocatalysis with TiO_2_ in suspension, both carried out in a Solarbox. This application enables evaluation of such processes according to their risk.

## 2. Results and Discussion

The environmental impact indexes are calculated as shown in Table 1, where the operations involved in the evaluation are given on the left, and the points evaluated are at the top. As shown in the second column, three types of operations are considered: normal, abnormal and emergency. The operations included in each group are described in the third column. Photocatalytic treatment of metoprolol tartrate salt (MET), a common β-blocker found in wastewater [12], in aqueous solution with TiO_2_ in suspension in the Solarbox, is presented as an example. In this case, the operations included are (see third column in Table 1):

- Normal: Preparation of MET solution, Weighting TiO_2_, Experiment Development, Sampling, Analysis, Reactor emptying and cleaning.
- Abnormal: Disassembly of experimental equipment for maintenance.
- Emergency: Tank or reactor breakdown and/or solvent spillage.

Two aspects are evaluated—pollution and consumption (second row in Table 1)—and each includes several specific parameters. Four parameters are considered for pollution: wastewater, solid waste, air emission and soil pollution (third row in Table 1). Consumption includes raw materials (in our case, MET), water and energy (third row in Table 1). For each of these (fourth row in Table 1), frequency (A), hazard (B) and amount (C) are considered. The final result, which is calculated as the product of these values, as shown in Column R*_ij_*, gives an idea of the risk of that aspect. Numerical values assigned to evaluate each item are from the criteria in Table 2, Table 3 and Table 4. The values proposed by Arteche [10] are taken as a first approximation. However, they have been modified and updated to current legislation and adapted to laboratory, pilot plant or industrial scale for this study.

**Table 1:** Environmental Risk Identification and Evaluation. Process tested: MET treatment by photocatalysis with TiO_2_ in the Solarbox. Parameters A–C are defined in Table 2, Table 3 and Table 4, respectively.

			Environmental Aspects																													
			Pollution																Consumption													
			1. Waste Water				2. Solid Waste				3. Air Emissions				4. Soil Pollution				5. Raw Materials				6. Water				7. Energy					
			A	B	C	Rij	A	B	C	Rij	A	B	C	Rij	A	B	C	Rij	A	B	C	Rij	A	B	C	Rij	A	B	C	Rij	E_Oi_	%E_Oi_, _max_
**Operation**	Normal Operation	1. Preparation of MET solution									MET powders								MET				Water									
											6	1	1	6					6	1	1	6	6	2	1	12					24	0.42
		2. Weighting of TiO_2_									TiO_2_ powders								TiO_2_													
											6	1	1	6					6	1	1	6									12	0.21
		3. Experiment Development					Gloves																				Electricity for lamp, pumps, stirrer					
							6	1	1	6																	6	5	2	60	66	1.16
		4. Sampling					Filters																									
							6	1	2	12																					12	0.21
		5. Analysis	Aqueous solution with organics								Air TOC								Reagents				Water for preparations				Electricity for equipment					
			6	10	3	180					6	5	2	60					6	10	2	120	6	2	1	12	6	5	3	90	462	8.11
		6. Reactor emptying and cleaning	Aqueous solution with organics				Adsorbent paper																Water for cleaning				Electricity for pump, stirrer					
			6	3	4	72	6	1	1	6													6	2	2	24	6	5	2	60	162	2.84
	Abnormal Operation	7. Disassembly of experimental equipment for maintenance					Crashing of reactor																									
							1	1	1	1																					1	0.02
	Emergency Operation	8. Tank or reactor breakdown and/or solvent spillage	Aqueous solution with organics				Adsorbent material																									
			1	3	2	6	1	1	1	1																					7	0.12
		E_Ai_	258				26				72				0				132				48				210				E = 746	-
		%E_Ai,max_	3.23				0.33				0.90				0				1.65				3.00				5.25				-	-

**Table 2:** Parameter A (Frequency): Numerical values for Parameter A, assigned by the frequency (probability) of occurrence of each event.

		Aspect to Be Evaluated						
		Waste-water	Solid Wastes	Air Emission	Soil Pollution	Raw Material	Water	Energy
**Frequency**	*Accidental*	1	1	1	1	1	1	1
	*Yearly*	2	2	2	2	2	2	2
	*Monthly*	4	4	4	4	4	4	4
	*Every two weeks*	5	5	5	5	5	5	5
	*Weekly*	6	6	6	6	6	6	6
	*Three times a week*	8	8	8	8	8	8	8
	*Daily*	10	10	10	10	10	10	10

**Table 3:** Parameter B (Hazard): Numerical values for Parameter B, assigned by how hazardous each event is.

Emission or Consumption Hazard	Rate	Notes
*Wastewater*		
No contact with raw materials or products	1	
Contact Non-hazardous	3	(1),(3)
Contact with Products with signal word Warning	6	(1),(3)
Contact with Products with signal word Danger	10	(1),(3)
*Solid Wastes*		
Not in Decision 2000/532/EC	1	(2),(3)
Not hazardous in Decision 2000/532/EC	5	(2),(3)
Hazardous in Decision 2000/532/EC	10	(2),(3)
*Air Emissions*		
Non-hazardous	1	(1),(3)
Products with signal word Warning	5	(1),(3)
Products with signal word Danger	10	(1),(3)
*Soil Pollution*		
Non-hazardous	1	(1),(3)
Products with signal word Warning	5	(1),(3)
Products with signal word Danger	10	(1),(3)
*Raw Materials*		
Non-hazardous	1	(1),(3)
Products with signal word Warning	5	(1),(3)
Products with signal word Danger	10	(1),(3)
*Water*		
Any water consumption	2	
*Energy*		
Any energy consumption	5	

(1) Classification according to CLP Regulation Signal Word. Regulation (EC) No 1272/2008 aligns existing EU legislation to the United Nations Globally Harmonised System (GHS). This new regulation on classification, labelling and packaging (“CLP Regulation”) contributes to the GHS aim for the same hazards to be described and labelled in the same way worldwide [13]; (2) Decision 2000/532/EC establishes a list of hazardous wastes [14,15]; (3) When there is a mix of products, if one of them has the signal word Danger, the score assigned is 10. If more than two products have one hazard level, the value assigned is the one corresponding to the next level. For instance, if more than two products have the signal word Warning, the value assigned is also 10 (corresponding to the signal word Danger).

**Table 4:** Parameter C (Amount): Numerical values for Parameter C, assigned by the amount of product or energy consumption involved.

Discharge/Emissions or Consumption	Rate
*Wastewater*	
<0.001 m^3^/year	1
0.001–0.01 m^3^/year	2
0.01–0.1 m^3^/year	3
0.1–1 m^3^/year	4
1–10 m^3^/year	5
10–100 m^3^/year	6
100–1000 m^3^/year	7
1000–10,000 m^3^/year	8
10,000–100,000 m^3^/year	9
>100,000 m^3^/year	10
*Solid Wastes*	
<0.001 tn/year	1
0.001–0.01 tn/year	2
0.01–0.1 tn/year	3
0.1–1 tn/year	4
1–10 tn/year	5
10–100 tn/year	6
100–1000 tn/year	7
1000–10,000 tn/year	8
10,000–100,000 tn/year	9
>100,000 tn/year	10
*Air Emission (Polluted Air Flow-Rate)*	
<1 Nm^3^/year	1
1–10 Nm^3^/year	2
10–100 1Nm^3^/year	3
100–1000 Nm^3^/year	4
10^3^–10^4^ Nm^3^/year	5
10^4^–10^5^ Nm^3^/year	6
10^5^–10^6^ Nm^3^/year	7
10^6^–10^7^ Nm^3^/year	8
10^7^–10^8^ Nm^3^/year	9
>10^8^ Nm^3^/year	10
*Soil Pollution (Amount of Product Discharged)*	
<0.001 tn/year	1
0.001–0.01 tn/year	2
0.01–0.1 tn/year	3
0.1–1 tn/year	4
1–10 tn/year	5
10–100 tn/year	6
100–1000 tn/year	7
1000–10,000 tn/year	8
10,000–100,000 tn/year	9
>100,000 tn/year	10
*Raw Materials*	
<0.001 tn/year	1
0.001–0.01 tn/year	2
0.01–0.1 tn/year	3
0.1–1 tn/year	4
1–10 tn/year	5
10–100 tn/year	6
100–1000 tn/year	7
1000–10,000 tn/year	8
10,000–100,000 tn/year	9
>100,000 tn/year	10
*Water*	
<0.1 m^3^/year	1
0.1–1 m^3^/year	2
1–10 m^3^/year	3
10–100 m^3^/year	4
100–1000 m^3^/year	5
1000–10,000 m^3^/year	6
10,000–100,000 m^3^/year	8
>100,000 m^3^/year	10
*Energy*	
<50 kW.h/year	1
50–500 kW.h/year	2
500–2500 kW.h/year	3
2500–15,000 kW.h/year	5
15,000–75,000 kW.h/year	7
75,000–150,000 kW.h/year	9
>150,000 kW.h/year	10

As an example, the proposed method has been applied to the photocatalytic treatment of MET with TiO_2_ in suspension in a Solarbox (Table 1). For each operation and each environmental aspect involved in the process, a R*_ij_* index can be evaluated, where subscript *i* indicates the operation and subscript *j* indicates the environmental aspect. In our case, *i* varies from 1–8, because eight operations are involved in the process (see third column in Table 1). On the other hand, subscript *j* varies from 1–7, because there are seven environmental aspects (see third row in Table 1). The specific agent is shown in the cell corresponding to each operation and for each environmental aspect. For instance, in Environmental Aspect 1 (wastewater), the specific wastewater generated is shown in the corresponding cells (see Table 1): *aqueous solution with organics* for Operation 6 (Reactor emptying and cleaning) and *aqueous solution with organics* for Operation 8 (Tank or reactor breakdown and/or solvent spillage).

Data related to energy or raw materials and reagent consumption are required for any environmental impact assessment. Electricity consumption data for photocatalytic treatment of MET in the Solarbox are shown in Table 5. Obviously, the consumption of raw materials must be known. This is summarized in Table 6. The consumption of reagents for analysis must also be included in raw materials consumption. These data are presented in Table 7.

**Table 5:** Electricity consumption in MET photocatalytic treatment experiments in a Solarbox for 50 experiments/year.

Experiment				
*Equipment*	*Time Use Equipment (h/exp)*	*Power (kW)*	*Consumption (kWh)*	*Yearly Consumption (kWh/y)*
Lamp (1000 W)	5.5	1.00	5.50	275
Pump (250–500 W)	5.5	0.40	2.20	110
Thermostatic bath (240 W at 20 °C)	5.5	0.24	1.32	66
Stirrer (1–5 W)	5.5	0.003	0.02	0.8
**Total consumption/year (kWh/y)**				452
**Analysis**				
*Equipment*	*Time Use Equipment (h/exp)*	*Power (kW)*	*Consumption (kWh)*	*Yearly Consumption (kWh/y)*
HPLC (12 samples × 15 min/sample)	3	2.50	7.50	375
TOC (12 samples × 15 min/sample)	3	2.20	6.60	330
Spectrophotometer (DQO+Fe+H_2_O_2_+SUVA)	0.2	0.25	0.05	2.5
Water deionization device (preparation 1 L water)	0.1	0.10	0.01	0.5
**Total consumption/year (kWh/y)**				708

**Table 6:** Reagent consumption in the photocatalytic experiments with a 1 L reaction volume for 50 experiments/year.

Reagents	Concentration—Amount/Exp	Total Amount/Exp	Yearly Consumption (kg)
Metoprolol tartrate salt	50 mg/L	50 mg	0.003
TiO_2_	0.4 g/L	0.4 g	0.02
Millipore Water		1.00 L	50

An explanation for one of the lines in Table 1 is given below to clarify its use (see shaded cells). For instance, for *Reactor emptying and cleaning* (Operation 6), subscript *i* (1–8) in the R*_ij_* index is 6. Wastewater is generated in this operation, which implies that subscript *j* (1–7) in R*_ij_* is 1. Wastewater frequency is weekly, and according to Table 2 criteria, a value of 6 can be assigned to Parameter A. Considering that it is an aqueous solution in contact with non-hazardous products, a value of 3 is assigned to parameter B, according to Table 3 criteria. Finally, the amount discharged is from 100 to 1000 L/year and a value of 4 is assigned to Parameter C, according to the criteria in Table 4. Thus, risk R*_ij_* (R*_61_*) associated with Operation 6 and wastewater discharged has a value of 72 (product of AxBxC). Similarly, for the same operation (Operation 6), the risk for water consumption (Aspect 6) is R*_66_*_,_ and the value for Parameter A is 6 (weekly frequency). For Parameter B, the value is 2 (water consumption hazard). Finally, Parameter C is 2 (between 100 and 1000 L/year). Thus, R*_66_* = 24. The rest of the risk indexes associated with *reactor emptying and cleaning* (Operation 6) can be calculated in a like manner.

**Table 7:** Reagent consumption in the analysis of photocatalytic experiments for 50 experiments/year.

Parameter	Amount/Sample	Samples/Exp	Total Amount/Exp	Yearly Consumption
*MET analysis*				
Acetonitrile HPLC	2.55 mL	15	38.25 mL	1.5 kg
Acidified water HPLC	10.2 mL	15	153.00 mL	7.7 kg
*TOC analysis*				
Synthetic air TOC (150 mL/min × 15/min/sample)	2250 mL	15	33750 mL	1.5 Nm^3^
*COD analysis*				
Dichromate COD	1.5 mL	4	6 mL	0.3 kg
H_2_SO_4_ COD	3.5 mL	4	14 mL	0.7 kg
*BOD*				
Reagents	Negligible	0		0
Lyophilized capsules	0.0625 capsules	4	0.25	12.5 capsules
*Toxicity*				
Osmotic adjuster	0.25 mL	6	1.5 mL	0.1 kg
Dilution water	7.5 mL	6	45 mL	2.3 kg
Bacteria restorative (1 mL/container)	0.056 mL	6	0.333 mL	0.02 kg
Bacteria	0.056 containers	6	0.333 cont.	17 containers
Sampling				
Filters for samples	1 filter	15	15 filters	750 filters

Except for acetonitrile (density = 786 kg/m^3^), solution density was considered to be 1000 kg/m^3^ because they are very diluted aqueous solutions.

All the R*_ij_* values in Table 1 were calculated as described in the examples above. When they have all been determined, the environmental risk index E_Oi_ associated with each operation, or the environmental risk index E_Aj_ associated with each environmental aspect, can be calculated. Thus, the second-last column in Table 1 shows the environmental risk associated with each operation E_Oi_, calculated as the sum of the risks:

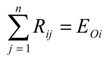
(1)


As an example, total risk E_O6_ associated with Operation 6 can be calculated as the sum of the risks associated with each aspect:

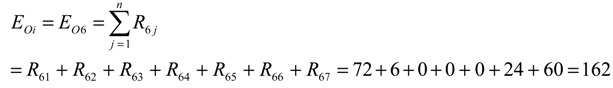
(2)


**Figure 1:**
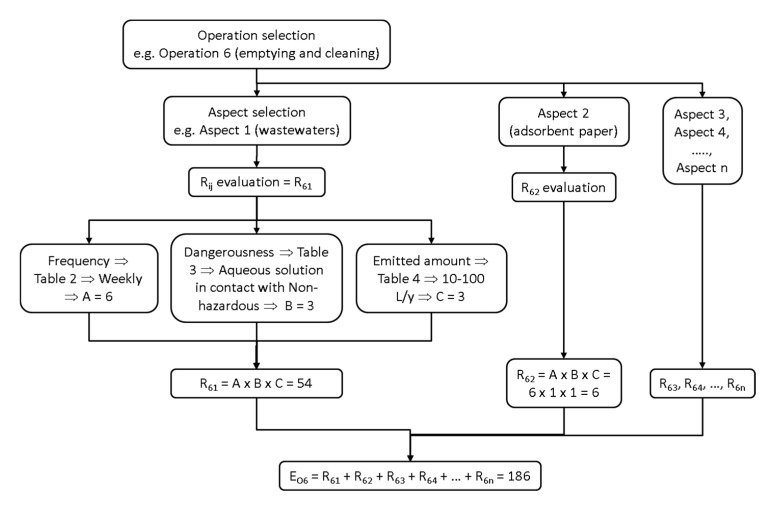
Evaluation of Environmental Risk Index: Schematic view.

This process is summarized schematically in Figure 1.

The last row in Table 1 shows the environmental risk associated with each environmental aspect E_Ai_, calculated as the sum of the risks for all the operations included in the process:

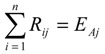
(3)


For example, for wastewater (see Environmental Aspect 1), the E_Ai_ is:

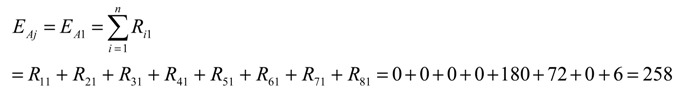
(4)


The Global Environmental Risk Index (E) for this process is the sum of the E_Oi_ or E_Aj_ values. This index appears in the second-to-the-last cell (bottom-right) in Table 1:

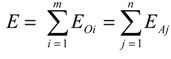
(5)


Applying all of this to the values in Table 1 results in:

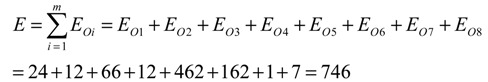
(6)


Or

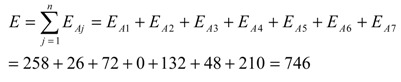
(7)


An interesting index is the percentage of risk for the maximum possible (%E_Oi,max_). The maximum R*_ij_* possible for each of the first five aspects (wastewater, solid wastes, air emission, soil pollution, raw materials consumption), is 1000, which is the product of the maximums possible for A, B and C, according to Table 2, Table 3 and Table 4. For water consumption, the maximum possible is 200 and for energy consumption the maximum is 500. Thus, the E_Oi_ maximum for any operation is 5700. Dividing 5700 by the E_Oi_ for each operation and multiplying by 100, %E_Oi,max_ can be found for each operation (see the last column of Table 1).

Similar reasoning follows for E_Ai,max_. Thus, the maximum E_Ai_ for each aspect is 8000 for the first five aspects (wastewater, solid waste, air emission, soil pollution, raw materials consumption), that is, 1000 for each aspect (10 for frequency, 10 for hazard and 10 for the amount) multiplied by the eight operations in our case. E_Ai,max_ is E_Ai_ × (100/8000). The maximum of E_Ai_ for water consumption is 1600. Thus, E_Ai,max_ for water consumption is 3.0%. For energy consumption, the maximum is 4000 and E_Ai,max_ is 5.25.

The environmental risk indexes can be found the same way for photo-Fenton MET treatment in a Solarbox. As in photocatalysis, first you need to know the electricity and raw materials consumed. The electricity consumption data are the same as for the photocatalytic experiments and the only change is the time needed for each experiment, which in this case is 3.5 h. Thus, the total consumption of electricity by year, corresponding to the experiments performed, is 288 kWh/y. The rest of the data are the same as in Table 5.

Data corresponding to reagent consumption appears in Table 8. Note that changes from Table 6 (photocatalysis) are TiO_2_ disappearance and FeSO_4_ and H_2_O_2_ appearance.

**Table 8:** Reagent consumption in photo-Fenton experiments with a 1-L reaction volume for 50 experiments/year.

Reagents	Concentration—Amount/Exp	Total Amount Reagent/Exp.	Yearly Consumption (Kg)
Metoprolol tartrate salt	50 mg/L	50 mg	0.003
FeSO_4_.7H_2_O (7 mg/L Fe)	7 mg/L	34.75 mg	0.002
H_2_O_2_ (100 mg/L)	100 mg/L	333.3 mg	0.017
Millipore Water		1.00 L	50

Neither reagents for analysis, nor filters for samples (Table 7) are needed in this case, while Fe and H_2_O_2_ analyses are. These changes from Table 7 (photocatalysis) are observed in Table 9 (photo-Fenton).

The risk indexes for photo-Fenton experiments can be evaluated using the amounts shown in the tables above, similar to what was explained for photocatalysis. Results are presented in Table 10.

Comparisons can be made by taking the value found for each operation in each process. For the two processes studied (photocatalysis and photo-Fenton), the operations with the most risk are those related to analysis (Operation 5 in Table 1 and Operation 6 in Table 10) due to the use of highly hazardous reagents. In both cases, Operation 7, related to reactor emptying and cleaning, is the second most risky, which seems logical because wastewater generated may contain hazardous products. Finally, the third position in this virtual ranking of environmental risk of operations is running the experiment (Operation 3 in Table 1 and Operation 4 in Table 10). For the seven aspects analysed in each operation, photo-Fenton values are usually higher. There are two reasons for that, the number of operations involved is higher and the products used in photo-Fenton are more hazardous than those used in photocatalysis.

**Table 9:** Reagent consumption in photo-Fenton experiment analyses for 50 experiments/year.

Parameter	Amount/Sample	Samples/Exp	Total Amount/Exp	Yearly Consumption
*MET analysis*				
Acetonitrile HPLC	2.55 mL	15	38.25 mL	1.5 kg
Acidified water HPLC	10.2 mL	15	153 mL	7.7 kg
*TOC analysis*				
Synthetic air TOC (150 mL/min × 15/min/sample)	2250 mL	15	33750 mL	1.5 Nm^3^
*COD analysis*				
Dichromate COD	1.5 mL	4	6 mL	0.3 kg
H_2_SO_4_ COD	3.5 mL	4	14 mL	0.7 kg
*H_2_O_2_ analysis*				
Reagents for H_2_O_2_ determination	1.5 mL	2	3 mL	0.15 kg
*Fe analysis*				
Phenanthroline for Fe determination	1.0 mL	2	2 mL	0.1 kg
*BOD*				
Reagents	Negligible	0	0	0
Lyophilized capsules	0.0625 capsules	4	0.25 capsules	12.5 capsules
*Toxicity*				
Osmotic adjuster	0.25 mL	6	1.5 mL	0.1 kg
Dilution water	7.5 mL	6	45 mL	2.3 kg
Bacteria restorative (1 mL/container)	0.056 mL	6	0.333 mL	0.02 kg
Bacteria	0.056 containers	6	0.333 cont.	17 containers

Except for acetonitrile (density = 786 kg/m^3^), solution density is considered to be 1000 kg/m^3^ because they are very diluted aqueous solutions.

Of course, when comparisons are made, it has to be taken into account that the absolute values of E depend on the number of operations involved in the process, and it seems logical for E to increase when the number of operations is higher. Thus, from the results of Table 1 and Table 10, photo-Fenton is more dangerous than photocatalysis because their risk indexes (E = E_Oi_ = E_Aj_) are 994 and 746, respectively.

However, the ratio of E to the number of operations (E/NOP) can be instructive with respect to process risk and a good parameter for processes comparison, because it represents an average. In addition, this ratio gives information on how hazardous process operations are. Of course, high E/NOP ratios imply more risky operations. The same may be said of the ratio of E to the number of aspects in the process (E/NAS). There are always seven aspects analysed in the process, as seen in Table 1 and Table 10. Thus, an increase in the E/NAS ratio means that the process environmental hazard increases. The E/NOP and E/NAS ratios are the highest for photo-Fenton (see Table 11).

**Table 10:** Environmental Risk Identification and Evaluation. Process tested: photo-Fenton MET treatment in the solarbox.

			Environmental Aspects																													
			Pollution																Consumption													
			1. Waste water				2. Solid Wastes				3. Air Emission				4. Soil Pollution				5. Raw Materials				6. Water				7. Energy					
			A	B	C	R_ij_	A	B	C	R_ij_	A	B	C	R_ij_	A	B	C	R_ij_	A	B	C	R_ij_	A	B	C	R_ij_	A	B	C	R_ij_	E_Oi_	%E_Oi,__max_
**Operation**	Normal Operation	1. Preparation of MET solution									MET powders								MET				Water									
											6	1	1	6					6	1	1	6	6	2	1	12					**24**	**0.42**
		2. FeSO_4_ addition																	FeSO_4_													
																			6	5	1	30									**30**	**0.53**
		3. H_2_O_2_ addition																	H_2_O_2_													
																			6	10	1	60									**60**	**1.05**
		4. Experiment Development					Gloves																				Electricity for lamp, pumps, stirrer					
							6	1	1	6																	6	5	2	60	**66**	**1.16**
		5. Sampling																														
																															**0**	**0**
		6. Analysis	Aqueous solution with organics								Air TOC								Reagents				Water for preparation				Electricity for equipment					
			6	10	3	180					6	5	2	60					6	10	2	120	6	2	1	12	6	5	3	90	**462**	**8.11**
		7. Reactor emptying and cleaning	Aqueous solution with organics				Adsorbent paper																Water for cleaning				Electricity for pump, stirrer					
			6	10	4	240	6	1	1	6													6	2	2	24	6	5	2	60	**330**	**5.79**
	Abnormal Operation	8. Disassembly of experimental equipment for maintenance					Crashing of reactor																									
							1	1	1	1																					**1**	**0.02**
	Emergency Operation	9. Tank or reactor breakdown and/or solvent spillage	Aqueous solution with organics				Adsorbent material																									
			1	10	2	20	1	1	1	1																					**21**	**0.37**
		**E_Ai_**	**440**				**14**				**66**				**0**				**216**				**48**				**210**				**E = 994**	-
		**%E_Ai, max_**	**4.89**				**0.16**				**0.73**				**0**				**2.40**				**2.67**				**4.67**				-	-

**Table 11:** Summarized final basic parameters for the environmental comparison of the processes.

Process	E	E/NOP	E/NAS	Av. %E_Oi_ max	Av. %E_Ai_ max
Photocatalysis	746	93	107	1.64	2.05
Photo-Fenton	994	110	142	1.94	2.22

Another parameter that can give an idea of the risk involved in the tested process is the percentage with respect to the maximum possible to be achieved for each operation and aspect (see last row and last column in Table 1 and Table 10). The averages are presented in Table 11, for both items and processes. The first comment to be made is that in all cases the values are very low. This means that the hazard levels of the processes analysed are very low.

## 3. Experimental Section

The processes in the study are well known and have been previously described elsewhere [16,17,18]. However, a short description is presented here to make it easier for the reader to understand. Metoprolol tartrate salt (MET) (a model pollutant) was treated with photo-Fenton or photocatalysis with TiO_2_ in a solarbox, as described previously [17].

Experiments were carried out in a solarbox (from CO.FO.ME.GRA) with an Xe lamp (1000 W). The solution to be treated was prepared in a feed tank (1 L) and pumped into a tubular reactor located at the bottom of the solarbox in the axis of a parabolic mirror. From there, the suspension was continuously recirculated to the feed tank. The feed tank was continuously stirred and the temperature was kept constant using a thermostatic bath. The experimental devices used are described at length elsewhere [16,17,18].

The parameters and variables monitored and analysed during the experiments were: MET concentration, TOC, COD, BOD, toxicity, *etc.* (see Table 7 and Table 9).

MET removal was the same in both processes, but was degraded much faster by photo-Fenton than by photocatalysis (70 min and 180 min, respectively, for 90% MET removal), for experiments done with the same experimental equipment and under the same conditions. However, mineralisation was very similar in both processes. On the other hand, photocatalysis proved to be more energy efficient (mg of MET removal/kJ of useful light) than photo-Fenton. A detailed comparison of these two technologies for MET removal is available in a previous paper [17].

## 4. Conclusions

Several indexes have been estimated: absolute Environmental Risk Index (E_Oi_) associated with each of the unit operations involved in the process, absolute Environmental Risk Index (E_Aj_) related to the conditions of process running, and Global Environmental Risk Index (E) of the process studied. In addition, E/NOP and E/NAS relative indexes for evaluating the risk of each operation or aspect involved in the processes have been proposed. With these indexes, operations and processes, *etc.*, may be compared to find the one that is environmentally best. This procedure was developed for lab scale, but its results could be used for plant design or operation, simply by entering data from large AOP wastewater treatment plants. It is important to highlight that the key point of the procedure is the correct selection of operations involved in each process to be analysed.

It should also be mentioned that the lab-scale operations with the highest risk are the analytical protocols (reinforcing the idea of further R&D on these protocols, especially when applied to evaluate processes for improving environmental protection). In our case, photocatalysis seems to involve less environmental risk than photo-Fenton.
